# Temporal Horn Enlargements Predict Secondary Hydrocephalus Diagnosis Earlier than Evans’ Index

**DOI:** 10.3390/tomography8030115

**Published:** 2022-05-25

**Authors:** Paolo Missori, Sergio Paolini, Simone Peschillo, Cristina Mancarella, Anthony Kevin Scafa, Emanuela Rastelli, Stefano Martini, Francesco Fattapposta, Antonio Currà

**Affiliations:** 1 Department of Human Neurosciences, Neurosurgery, Policlinico Umberto I, “Sapienza” University of Rome, 00185 Rome, Italy; anthonykevin.scafa@uniroma1.it; 2 IRCCS Neuromed-Pozzilli, “Sapienza” University of Rome, 86077 Pozzilli, Italy; sergio.paolini@uniroma1.it (S.P.); cristina.mancarella@gmail.com (C.M.); 3 Department of Neurosurgery, University of Catania, 95124 Catania, Italy; simone.peschillo@gmail.com; 4 Department of Radiology, Neuroradiology, Policlinico Umberto I, “Sapienza” University of Rome, 00185 Rome, Italy; emanuela.rastelli@uniroma1.it (E.R.); stefano.martini@uniroma1.it (S.M.); 5 Department of Human Neurosciences, Neurology, Policlinico Umberto I, “Sapienza” University of Rome, 00185 Rome, Italy; francesco.fattapposta@uniroma1.it; 6 Academic Neurology Unit, Department of Medical-Surgical Sciences and Biotechnologies, Ospedale A. Fiorini, “Sapienza” University of Rome, 04019 Terracina, Italy; antonio.curra@uniroma1.it

**Keywords:** brain, haemorrhage, hydrocephalus, injury, temporal horn, ventricular system

## Abstract

The aim of this study was to identify early radiological signs of secondary hydrocephalus. We retrieved neuroradiological data from scans performed at various times in patients who underwent surgery for secondary hydrocephalus due to severe traumatic brain injury (TBI), subarachnoid haemorrhage (SAH), or brain tumour (BT). Baseline measurements, performed on the earliest images acquired after the neurological event (T0), included Evans’ index, the distance between frontal horns, and the widths of both temporal horns. The next neuroimage that showed an increase in at least one of these four parameters—and that lead the surgeon to act—was selected as an indication of ventricular enlargement (T1). Comparisons of T0 and T1 neuroimages showed increases in Evans’ index, in the mean frontal horn distance, and in the mean right and left temporal horn widths. Interestingly, in T1 scans, mean Evans’ index scores > 0.30 were only observed in patients with BT. However, the temporal horn widths increased up to ten-fold in most patients, independent of Evans’ index scores. In conclusion temporal horn enlargements were the earliest, most sensitive findings in predicting ventricular enlargement secondary to TBI, SAH, or BT. To anticipate a secondary hydrocephalus radiological diagnosis, clinicians should measure both Evans’ index and the temporal horn widths, to avoid severe disability and poor outcome related to temporal lobe damage.

## 1. Introduction

Acute or subacute secondary hydrocephalus can appear as a complication in the clinical course of some neurosurgical conditions. In these cases, because the patient can be unconscious or symptoms are not striking, the clinical diagnosis may be delayed. Hydrocephalus is evaluated on a computed tomography (CT) scan with Evans’ index, which is the ratio between the maximum width of the frontal horns and the maximal internal diameter of the skull at the level of Monro’s foramens. A radiological diagnosis of hydrocephalus is defined as an Evans’ index value > 0.30 [1]. However, this index value may not surpass the cut-off value in early radiological examinations, when enlargement is mild in the frontal horns.

To prevent damage to the periventricular temporal brain, it is particularly important to determine ventricular enlargements as early as possible, and consequently, to diagnosis hydrocephalus. In neuroimages, compared to the frontal horns, the temporal horns appear as two thin slits in the most distal portion of the ventricular system [2]. In post-traumatic hydrocephalus, marked temporal horn enlargement has been documented on magnetic resonance imaging (MRI) scans [3]. In children, the temporal horn ratio is routinely calculated to diagnose paediatric hydrocephalus [4,5]. In adults, idiopathic normal-pressure hydrocephalus in the ventricular system frequently causes more enlargement in the temporal horns than in the frontal horns [6,7]. Moreover, some studies have reported temporal horn enlargement in the diagnosis of acute hydrocephalus in patients with brain injuries or brain tumours (BTs) [8,9]. However, changes in the temporal horns in acute and subacute hydrocephalus have not been analysed in detail.

In the present study, by looking at brain scans of patients who had a clinical diagnosis of secondary hydrocephalus and underwent surgery, we aimed to identify the radiological signs of hydrocephalus. When the patient’s symptoms suggest incoming hydrocephalus, surgeons raise the suspicion of a secondary hydrocephalus. Thereafter, they search for a radiological confirmation, i.e., to ascertain that ventricular enlargement drives neurological deterioration. We investigated the concurrent changes in the Evans’ index, frontal horn distance, and temporal horn widths to see whether the latter might be an early and sensible marker of secondary hydrocephalus more than the Evan’s index. To that end, we studied serial CTs and MRIs from patients that developed acute or subacute ventricular enlargements after severe traumatic brain injury (TBI), subarachnoid haemorrhage (SAH), or BTs who underwent surgery based on a clinical diagnosis of secondary hydrocephalus.

## 2. Materials and Methods

After having obtained the informed consent from patients or next of kin, we conducted a retrospective review, IRB-approved (ref. 6630 9 February 2022) of head neuroradiological data (CT scan and/or MRI imaging) from November 2009 to June 2021. Participants were patients affected by severe TBI, SAH from a ruptured aneurysm, or BT, who underwent emergency surgical external cerebrospinal fluid diversion or early ventriculoperitoneal shunt for acute or subacute secondary hydrocephalus. CT or MRI scans at the first indication of ventricular enlargement and possibly hydrocephalus (defined as Evans’ index > 0.30 at Monro’s foramens) were defined as T1 scan; any available previous baseline neuroimage were considered T0 scan. Scans of patients that were dilated at first radiological imaging (T0) were excluded from the analysis. For each patient, the time (days) from T0 to T1 was recorded.

**Figure 1:**
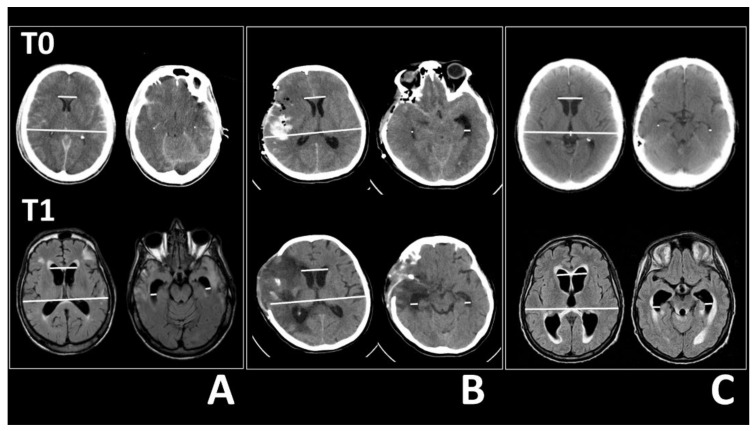
Images of CTs and MRIs acquired at admission (T0, *top row*) and at the first sign of ventricular enlargement (T1, *bottom row*) in three patients. (**A**) TBI patient, (**B**) SAH patient, (**C**) BT patient. In each panel, *left* images show the frontal horn distances and Evans’ index measurements (*white lines*), and *right* images show the temporal horn width measurements (*white lines*). (**A**) Compared to the CT images (T0), the MRI scans after 8 weeks (T1) shows that the frontal horns enlarged from 29.1 to 39.2 mm and the Evans’ index increased from 0.22 to 0.3 (*left*). The right temporal horn enlarged from 1.4 to 7.7 mm and the left temporal horn enlarged from 0.9 to 7.3 mm (*right*). (**B**) T0 image is scanned after clipping a middle right cerebral artery aneurysm and performing a decompressive craniectomy for SAH. A CT image acquired 19 days after surgery (T1) shows that the frontal horn distance enlarged from 30.1 to 37.7 mm and Evans’ index increased from 0.22 to 0.28 (*left*). The right temporal horn width enlarged from 1.9 to 9.1 mm and the left temporal horn width enlarged from 7.7 to 8.2 mm (*right*). (**C**) T0 CT scan shows a patient with fourth ventricle lymphoma. After 3 weeks (T1), the MRI shows an enlarged frontal horn distance (from 33.6 to 43.5 mm) and an increase in Evans’ index, from 0.25 to 0.32 (*left*). The right temporal horn width enlarged from 1.3 to 10.9 mm and the left temporal horn width enlarged from 1.7 to 11.4 mm (*right*).

Two independent observers (one neuroradiologist and one neurosurgeon with long neurosurgical experience on secondary hydrocephalus and its surgical treatment) examined axial scans and calculated Evans’ index, the bifrontal horn distance, which had been used to calculate Evans’ index, and the widths of the right and left temporal horns, strictly measured at the level of the ventricular prominence of the hippocampus (Figure 1). Agreement between observer measures was evaluated with the Kappa statistic. The percentage change from T0 to T1 was calculated for each measure (*M*) with the formula: (*M*T1 − *M*T0)/*M*T0 × 100%.

For each radiological measure, the raw data and the percentage change were analysed with a multivariate, repeated measures analysis of variance (ANOVA), to compare repeated measures over time (TIME) and between conditions (CONDITION; i.e., mode of brain injury). The changes in time, from T0 to T1 (days), were analysed with a univariate ANOVA. Post-hoc analysis was performed with the Tukey Honest Significance Difference test and the Bonferroni correction for multiple comparisons when needed. Statistical significance was set to *p* < 0.05.

## 3. Results

We found a total of 234 patients who underwent temporary external or immediate shunt for acute or subacute secondary hydrocephalus. One hundred and twelve patients had no available scan in the database, due to missing transfer from a previously operating system archive. The remaining 122 patients had previous neuroimages scanned at varying time intervals (T0 and T1). Among these patients, 11 were excluded from the analysis because the ventricular system was already dilated at T0 (therefore at T0 all patients had Evans’ index < 0.30) and 36 for unclear ventricular measurements due to inaccurate tilt of patient’s head. Finally, we included data on 75 patients (145 CT scan: 97%, 5 MRI: 3%), including 30 with TBIs (mean age: 51 ± 16.0 years, 21 men), 30 with SAH (mean age: 59.5 ± 15.7 years, 21 women), and 15 with BTs (mean age: 59 ± 15.2 years, 12 women; 10 posterior fossa tumours and 5 supratentorial tumours). The comparison between observer CT or MRI evaluations resulted in a Kappa value of 0.91. This finding indicated almost perfect agreement between observers in evaluating the Evans’ index and the frontal and temporal horn measurements.

**Figure 2:**
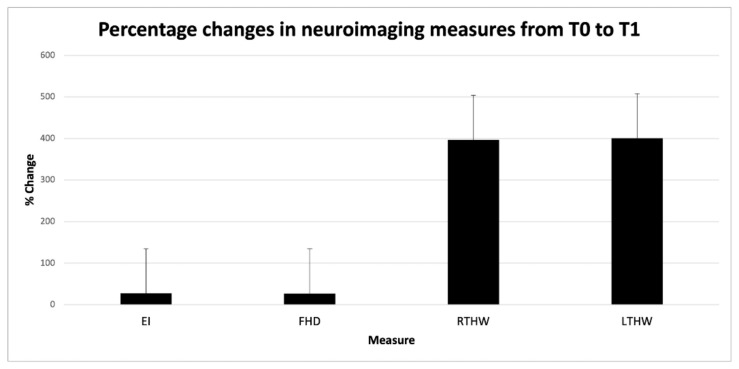
The percentage changes were greater in the right and left temporal horns (RTHW and LTHW) than in Evans’ index (EI) and the frontal horn distance (FHD).

A multivariate, repeated measures ANOVA of Evans’ index, the frontal horn distance, and the right and left temporal horn widths showed a significant effect of TIME (F = 35,669, *p* < 0.001), but not CONDITION (F = 1946, *p* = 0.056). These findings indicated that all measures changed from T0 to T1, independent of the type of brain disease. The multivariate ANOVA of the percent changes in the Evans’ index (27.5%), the frontal horn distance (26.8%), and the right (394.3%) and left temporal horn (398.3%) widths showed that the percentage changes were greater in the right and left temporal horns than in Evans’ index and the frontal horn distance (Figure 2).

**Figure 3:**
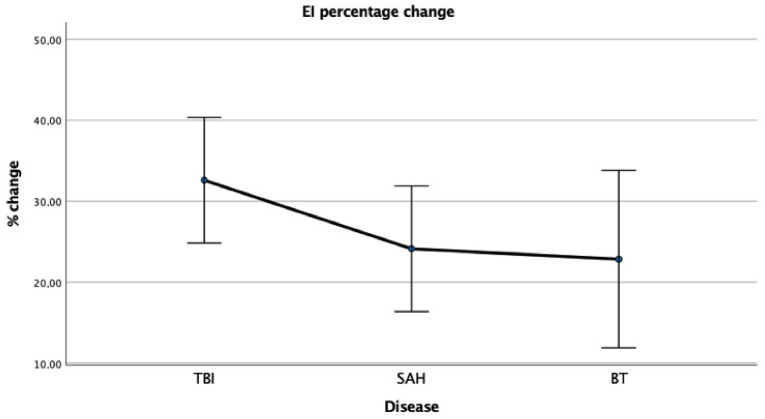
Percentage changes in the Evans’ index (EI) in the three study groups. Bars indicate Standard deviations.

The percentage change in EI for each group of patients are displayed in Figure 3.

A univariate ANOVA of the T0–T1 time interval showed that the differences in the time to a neuroimaging follow-up did not reach statistical significance between patients with different conditions (F = 0.648, *p* = 0.526). TBI patients had mean T0–T1 interval of 54.4 ± 56.3 days; SAH patients had mean T0–T1 interval of 48.9 ± 71.4 days; BT patients had mean T0–T1 interval of 72.7 ± 76.5 days.

At T1, the mean Evans’ index was >0.30 only in patients with BT (Table 1). When we analysed the conditions separately, we found that, at T1, the Evans’ indexes were >0.30 in 13/30 patients with severe TBI (**43%**), in 11/30 patients with SAH (**36%**), and in 10/15 patients with BTs (**67%**). These findings mean that 41 out 75 patients underwent surgery based on the surgeon’s clinical judgement that although not suprathreshold, the ventricular enlargement seen on brain images was driving the patient neurological deterioration.

Among patients with Evans’ indexes < 0.30 (i.e., who had radiological evidence of ventricular enlargement but not suprathreshold value of Evans’ index), we found that the frontal and right and left temporal horn measurements showed significant increases in all groups (*p* < 0.05). Percentage changes in frontal horn distance, right temporal horn width, and left temporal horn width are reported for these patients (Table 2 rows 2–4), along with those of patients having both clinical diagnosis and suprathreshold value of Evans’ index (Table 2, rows 6–8).

## 4. Discussion

This study demonstrated that CT- and MRI scan measurements of Evans’ index, frontal horn distance, right temporal horn width, and left temporal horn width increased in patients who underwent surgery for secondary hydrocephalus. We found that in these patients the temporal horns enlarged enormously—much more than the frontal horns. Indeed, the Evans’ index and frontal horn distance increased approximately 33% between the T0 and T1 scans, during the same period that the temporal horn widths doubled, tripled, or even quadruplicated. Therefore, temporal horn enlargement contributes to early diagnosis of secondary hydrocephalus earlier than the Evans’ index. Indeed, at T1 less than half patients (n = 34, 45%) who received surgery also had a suprathreshold value of the Evans’ index (i.e., they had a radiological diagnosis of secondary hydrocephalus). Most of these patients had brain tumours, and we reason that such a prevalent enlargement of the frontal horns is likely to be due to two factors. First, BT patients undergo neuroimaging evaluation later in the course of disease than patients with SAH and TBI patients. Second, they show longer time intervals between consecutive neuroimaging evaluations than SAH and TBI patients, as also suggested by statical analysis of T0–T1 intervals in the three study groups. In contrast, all 75 patients had the temporal horns significantly enlarged, most extensively the TBI group, possibly because of faster development of secondary hydrocephalus coupled with a less frequent neuroradiological evaluation. Overall, more than one patient out two (41 out 75, 55%) underwent emergency surgery based on the clinical judgement that huge temporal enlargement was driving the patient’s neurological deterioration.

We observed a similar disproportionate temporal horn enlargement in 27 other patients that evolved to hydrocephalus secondary to malignant ischemia, intraparenchymal haemorrhage, infectious diseases, meningeal carcinomatosis, and aqueductal stenosis; these patients were not included in the present analysis, due to the very small sample sizes for each of these conditions.

Findings of this neuroimaging study suggest that clinicians should not adopt a wait-and-see management strategy for patients that show temporal horn enlargement, but normal Evans’ index values. That strategy is likely to increase the risk that unconscious or drowsy patients might undergo irreversible brain damage in the temporal periventricular neural tissue. Indeed, the frontal horns can increase to about 33% above their initial value (Figure 2) without necessarily reaching an Evans’ index > 0.3. During the time spent waiting for a second brain scan, the temporal horns can enlarge to maximum sizes. The temporal horns are surrounded by the hippocampus ventro-medially and the amygdala supero-medially; the tail of the caudate nucleus and the optic radiation form the roof of the horns. When untreated, marked enlargement of the temporal horns disrupts neurogenesis in the dentate gyrus from multipotent, self-renewing progenitor cells of neurons and glial cells, can impair cognitive functions, such as learning and memory abnormalities, and contribute to executive dysfunction. Temporal horn enlargements can also cause seizures (temporal lobe epilepsy) and favour contralateral homonymous hemianopia and contralateral hemiparesis (indirectly, by compressing the internal capsule) [10,11,12]. Persistent temporal horn dilatations were previously reported to cause flattening of the hippocampal formations, due to neural tissue loss [2]. In such cases severe disability and poor outcome related to temporal lobe damage result.

Conversely, when patients are rapidly treated with cerebrospinal fluid diversion, the temporal horn enlargement resolves. In particular, a fast shunting or endoscopic third ventriculostomy can immediately resolve temporal horn enlargements. Therefore, to prevent irreversible brain damage due to a delayed radiological diagnosis, temporal horn widths should be monitored in conjunction with the Evans’ index. Measuring both these neuroimaging parameters will facilitate the surgeon in the decision process, avoiding the embarrassing dilemma of brain atrophy vs. posttraumatic hydrocephalus, which has long challenged clinicians [13,14]. To confirm that cerebrospinal fluid drainage after cerebrospinal fluid diversion is adequate, the enlargement should be carefully checked also at a neuroimaging follow-up. 

A previous study examined 13 ventriculograms and encephalograms and reported that the normal temporal horn width, at the level of the lateral cleft, was 5.5–7.2 mm [15]. In other studies, a lateral cleft measurement that exceeded 2–4 mm was considered dilated [10,16,17]. In our experience, in examining neuroimaging scans from healthy paediatric and adult subjects, the temporal horn cavities at the hippocampus level have very rarely exceeded 1 mm in width. It is common to see increased temporal horn widths and frontal horn distance on neuroimaging scans in individuals with normal brain aging, and in patients with idiopathic normal-pressure hydrocephalus as well. Therefore, having a standardized norm that encourages to measuring this width would prove beneficial in many conditions causing ventricular enlargement.

This study, which is intended as a pilot study to lay the groundwork for a more complete research study in the future, has some limitations. First, lack of prior research studies on the topic, which presented us with the dilemma of the diagnostic criterion for patient recruitment. Diagnosis of secondary hydrocephalus is radiological, based on Evans’ index value > 0.30. Yet our retrospective investigation showed that surgeons operated even in the absence of this criterion. This observation prompted us to recruit patients on the basis of the clinical diagnosis (and the surgery having been performed) and not the radiological criterion, allowing us to highlight a radiological marker earlier than Evans’ index. Second, the retrospective nature of the study that introduced inherent limitations. The present sample size is small, and we know that statistical tests require large sample sizes. However, enrolment criteria constrained to collect clear and readable CT or MRIs scans at two time points in the clinical course of every patient. In our real-world setting, many scans were not available in the database, due to missing transfer from a previously operating system archive. In addition, rarity of secondary hydrocephalus due to TBI, SAH or BT further limited the number of patients enrolled. Many other patients with different pathologies or with evident secondary hydrocephalus at first examination (T0) were also excluded. Nevertheless, due to the clinical impact of the observation, we considered reasonable to convey the message that temporal horn enlargement is an early and sensitive marker of secondary hydrocephalus.

In conclusion, the series of scans presented in this study showed that temporal horn enlargement disproportionately outpaced frontal horn enlargement in many patients with TBI, SAH, or BT with manifest or imminent secondary hydrocephalus who underwent surgery. These results suggest that joint measures of frontal distances and temporal horn widths should be performed routinely in neuroimaging analyses to facilitate the rapid diagnosis and treatment of acute or subacute secondary ventricular enlargement (i.e., hydrocephalus).

## Figures and Tables

**Table 1:** Comparison of measurements between baseline (T0) and the first CT scan that showed an indication of ventricular enlargement (T1). (*) indicates *p* < 0.05 for each comparison (T0 vs. T1) in each row.

	Evans’ Index (Mean ± SD)		Frontal Horn Distance, mm (Mean ± SD)	
Condition	T0	T1	T0	T1
Traumatic Brain Injury	0.22 ± 0.03	0.30 ± 0.04 *	29.9 ± 4.14	39.2 ± 6.45 *
Subarachnoid haemorrhage	0.25 ± 0.03	0.30 ± 0.04 *	32.3 ± 4.70	39.1 ± 5.28 *
Brain tumour	0.26 ± 0.04	0.31 ± 0.03 *	33.6 ± 6.11	40.7 ± 4.91 *
	**Right Temporal Horn Width, mm (Mean ± SD)**		**Left Temporal Horn Width, mm (Mean ± SD)**	
Traumatic Brain Injury	1.3 ± 0.55	7.8 ± 4.43 *	1.2 ± 0.60	6.7 ± 4.26 *
Subarachnoid haemorrhage	1.8 ± 0.76	6.9 ± 2.77 *	2.4 ± 1.76	6.0 ± 3.94 *
Brain tumour	1.3 ± 0.44	5.0 ± 2.49 *	1.5 ± 0.62	6.3 ± 2.67 *

**Table 2:** At T1 mean percent increases in frontal and temporal horn measurements showing an indication of ventricular enlargement. All percentage differences are significant *p* < 0.05. Values are the mean (±standard deviation); TBI: traumatic brain injury; SAH: subarachnoid haemorrhage; BT: brain tumour.

Evans Index < 0.30	Mean Change in Frontal Horn Distance (%)	Mean Change in Right Temporal Horn Width (%)	Mean Change in Left Temporal Horn Width (%)
TBI (0.26 ± 0.02)	24.86 ± 15.83	432.82 ± 404.45	549.12 ± 583.30
SAH (0.27 ± 0.01)	17.82 ± 11.68	318.17 ± 212.03	130.43 ± 134.06
BT (0.27 ± 0.01)	19.96 ± 11.84	360.30 ± 155.52	543.34 ± 615.44
**Evans Index > 0.30**			
TBI (0.34 ± 0.03)	41.33 ± 27.19	683.92 ± 476.93	541.40 ± 388.37
SAH (0.34 ± 0.03)	26.62 ± 16.09	293.23 ± 199.25	328.76 ± 358.58
BT (0.33 ± 0.01)	22.90 ± 18.60	239.68 ± 193.90	324.99 ± 179.14

## Data Availability

The data presented in this study are available on request from the corresponding author.
